# Hypoxia-Induced *FAM13A* Regulates the Proliferation and Metastasis of Non-Small Cell Lung Cancer Cells

**DOI:** 10.3390/ijms22094302

**Published:** 2021-04-21

**Authors:** Iwona Ziółkowska-Suchanek, Marta Podralska, Magdalena Żurawek, Joanna Łaczmańska, Katarzyna Iżykowska, Agnieszka Dzikiewicz-Krawczyk, Natalia Rozwadowska

**Affiliations:** Institute of Human Genetics, Polish Academy of Sciences, Strzeszyńska 32, 60-479 Poznań, Poland; marta.podralska@igcz.poznan.pl (M.P.); magdalena.zurawek@igcz.poznan.pl (M.Ż.); joanna.laczmanska@gmail.com (J.Ł.); katarzyna.izykowska@igcz.poznan.pl (K.I.); agnieszka.dzikiewicz-krawczyk@igcz.poznan.pl (A.D.-K.); natalia.rozwadowska@igcz.poznan.pl (N.R.)

**Keywords:** non-small cell lung cancer, FAM13A gene, hypoxia, cell proliferation, cell migration, invasion

## Abstract

Hypoxia in non-small cell lung cancer (NSCLC) affects cancer progression, metastasis and metabolism. We previously showed that FAM13A was induced by hypoxia in NSCLC but the biological function of this gene has not been fully elucidated. This study aimed to investigate the role of hypoxia-induced FAM13A in NSCLC progression and metastasis. Lentiviral shRNAs were used for *FAM13A* gene silencing in NSCLC cell lines (A549, CORL-105). MTS assay, cell tracking VPD540 dye, wound healing assay, invasion assay, BrdU assay and APC Annexin V staining assays were performed to examine cell proliferation ability, migration, invasion and apoptosis rate in NSCLC cells. The results of VPD540 dye and MTS assays showed a significant reduction in cell proliferation after FAM13A knockdown in A549 cells cultured under normal and hypoxia (1% O_2_) conditions (*p* < 0.05), while the effect of FAM13A downregulation on CORL-105 cells was observed after 96 h exposition to hypoxia. Moreover, FAM13A inhibition induced S phase cell cycle arrest in A549 cells under hypoxia conditions. Silencing of FAM13A significantly suppressed migration of A549 and CORL-105 cells in both oxygen conditions, especially after 72 and 96 h (*p* < 0.001 in normoxia, *p* < 0.01 after hypoxia). It was showed that FAM13A reduction resulted in disruption of the F-actin cytoskeleton altering A549 cell migration. Cell invasion rates were significantly decreased in A549 FAM13A depleted cells compared to controls (*p* < 0.05), mostly under hypoxia. FAM13A silencing had no effect on apoptosis induction in NSCLC cells. In the present study, we found that FAM13A silencing has a negative effect on proliferation, migration and invasion activity in NSCLC cells in normal and hypoxic conditions. Our data demonstrated that FAM13A depleted post-hypoxic cells have a decreased cell proliferation ability and metastatic potential, which indicates FAM13A as a potential therapeutic target in lung cancer.

## 1. Introduction

Lung cancer imposes a major disease burden in the world as it remains the most commonly diagnosed cancer and the biggest cause of cancer-related deaths. Globally, lung cancer accounts for 17% and 9% of all cancers in men and women, respectively, and represents 19% of all cancer-related deaths. Although novel approaches for treating lung cancer are available, the 5-year survival rate is still low, ranging from 5 to 15% [1]. This is related to the advanced disease stage at diagnosis [2]. The major problem in lung cancer therapy is clinical resistance, in which hypoxia is one of the key components. Sustained hypoxia has been identified as an adverse indicator of patients’ prognosis independent of clinical stage at the moment of diagnosis [3].

Hypoxia (diminished oxygen availability) is the most common microenvironment feature of tumors and contributes significantly to their development. Hypoxia occurs in 90% of solid tumors due to limitations in diffusion and inefficient vasculature [3]. In the course of adaptation to hypoxia, tumors evolve more aggressive and therapy resistant phenotypes [4]. Oxygen deprivation induces many changes in gene expression, mainly through stabilization of the hypoxia-inducible subunits (HIFs) that regulate the cellular response to hypoxia. It is known that HIF-1 regulates thousands of genes [5]. However, there is a discrepancy in the predictive value of HIF-1 and its downstream genes expression, so there is a need for the development of gene expression signatures for assessing tumor hypoxia.

We previously showed that FAM13A was significantly up-regulated in lung cancer cell lines (A549, CORL-105) and non-small cell lung cancer fragments cultured ex vivo, after exposure to chronic hypoxia [6]. *FAM13A* (family with sequence similarity 13, member A, MIM 147582) gene encodes two main protein isoforms (1 and 2). Only isoform 1 contains the Rho-GAP domain that is important for the regulation of cell proliferation and survival [7]. It is known that Rho GTPases family proteins play an important role in the process of cell migration and several signaling complexes [8]. Perturbed Rho GTPase signaling is associated with several lung diseases, including lung cancer [9]. Previously, *FAM13A* gene variants were indicated as risk factors of lung cancer [10], chronic lung diseases including chronic obstructive pulmonary disease (COPD) [11,12] and cystic fibrosis (CF) [13]. Nevertheless, the biological function of FAM13A has not been fully elucidated and the mechanism of protein activity is poorly understood.

The aim of the present study is therefore to investigate the biological role of hypoxia-induced FAM13A in non-small cell lung cancer (NSCLC) progression. We examined the effect of FAM13A knockdown on cell proliferation, cell cycle, migration, invasion and apoptosis in NSCLC cells cultured under hypoxic conditions in order to clarify the function of FAM13A.

## 2. Results

### 2.1. Generating Lung Cancer Cell Lines with FAM13A Knockdown

**Figure 1:**
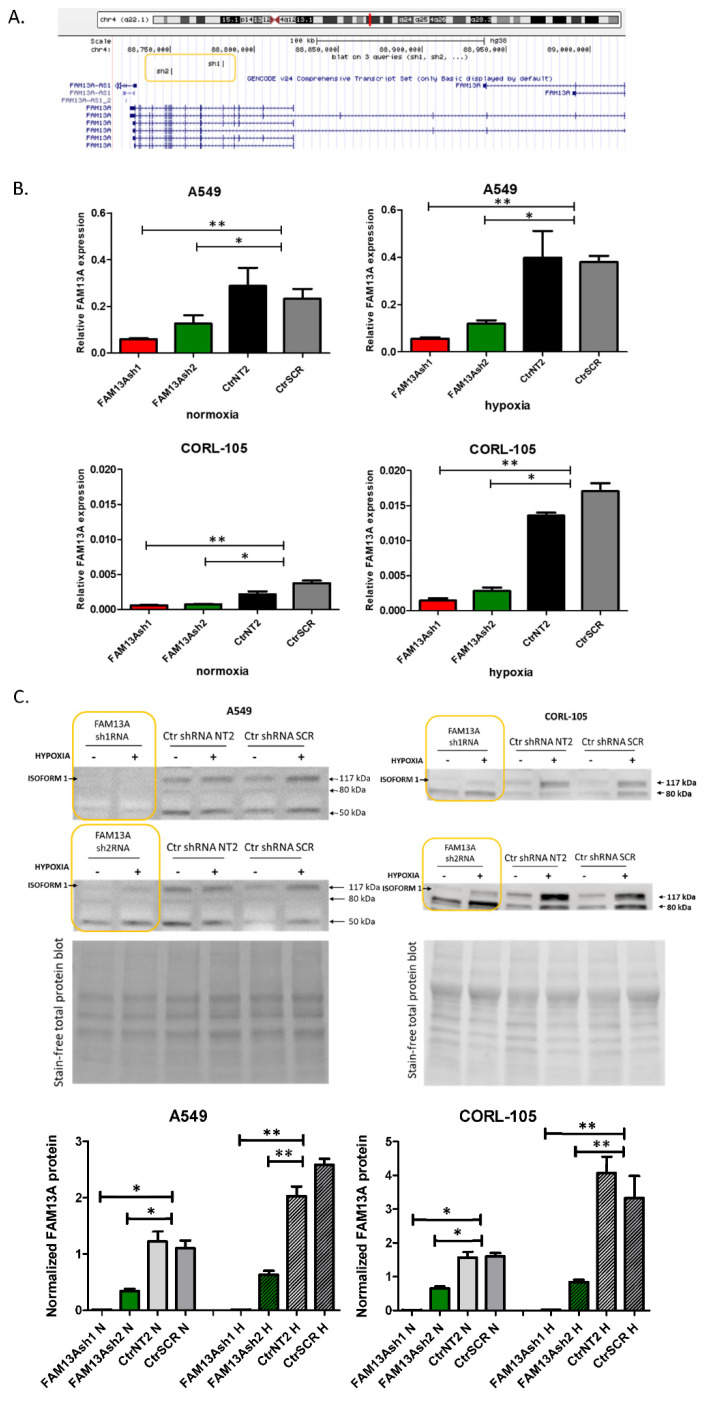
Generating lung cancer cell lines with FAM13A knockdown. FAM13A shRNAs (FAM13Ash1, FAM13Ash2) and control shRNAs (CtrNT2, CtrSCR) lentiviral particles were used to generate the stable transduction of A549 and CORL-105 cell lines. Transduction efficiency was assessed with the GFP marker. Knockdown of FAM13A mRNA and FAM13A protein was confirmed by real-time quantitative PCR and western-blot analysis. (**A**) Schematic indicating the binding site for the shRNA in the FAM13A mRNA. Two shRNA transcripts were designed using UCSC Broad Institute GPP Web Portal, against a coding sequence common to the most FAM13A isoforms with the RhoGAP functional domain. (**B**) RT-qPCR analysis of *FAM13A* expression in A549 and CORL-105 cell lines cultured under normoxia and hypoxia conditions for 72 h. Relative expression of FAM13A mRNA was determined as the mean normalized expression of FAM13A mRNA/GUSβ mRNA. The experiments were performed in triplicate and repeated three times. The data are presented as the mean ± SD (* *p* < 0.05, ** *p* < 0.01, unpaired one-tailed *t*-test). (**C**) Western blot (WB) analysis of FAM13A expression in A549 and CORL-105 cell lines cultured under normoxia and hypoxia for 72 h. Relative FAM13A protein (117 kDA) amount was significantly decreased in FAM13A sh1/sh2RNA cells. Strong FAM13A expression was induced in controls (CtrNT2, CtrSCR) A549 or CORL105 cells after 72 h of hypoxia (+) compared to normoxia (-). Cropped images are displayed, full-length blots are presented in Supplementary Materials Figure S4. Total protein was used for normalization. Representative stain-free total protein blots are presented. The normalized protein factors are given at bar charts (N-normoxia, H-hypoxia, bars with texture). The data are presented as the mean ± SD (* *p* < 0.05, ** *p* < 0.01, unpaired one-tailed *t*-test).

In our previous study, we have indicated that the expression of *FAM13A* was significantly up-regulated under hypoxia conditions in two lung cancer cell lines (A549, CORL-105, *p* < 0.001) and in lung cancer tissue fragments (*p* = 0.0004) [6]. In this study we decided to analyze the impact of FAM13A knockdown in lung cancer cell lines. *FAM13A* shRNAs (FAM13Ash1, FAM13Ash2) and control shRNAs (CtrNT2, CtrSCR) lentiviral particles were transduced into the A549 and CORL-105 cell lines (Figure 1A). Transduction efficiency was assessed with the GFP marker and it reached over 90% in both cell lines (Figure S1). Knockdown of *FAM13A* mRNA and FAM13A protein was confirmed by real-time quantitative PCR and western-blot analysis (Figure 1B,C), respectively. For FAM13A sh1RNA and FAM13A sh2RNA cells stable FAM13A knockdown was obtained, measured after 72 h of culture in both oxygen concentrations. Induction of *FAM13A* gene expression after hypoxia was observed among control cells (fold change FC, compared to normoxia): CtrNT2 (A549 FC = 2.2, CORL-105 FC = 2.1), CtrSCR (A549 FC = 2.5, CORL-105 FC = 2.8) and also wild-type cells (A549 FC = 2.2, CORL-105 FC = 2.1). Among shRNA treated cells this induction in hypoxia was strongly suppressed. During western blot analysis we detected the FAM13A isoform 1 with a molecular weight of about 117 kDa and the FAM13A isoform 2 (80 kDa). In A549 cells we observed the protein band with a molecular weight of about 50 kDa which could be the additional FAM13A isoform, as previously described [14]. Among shRNA treated cells specific downregulation of isoform 1 (117 kDa), containing the RhoGAP functional domain, was confirmed.

### 2.2. Silencing of FAM13A Inhibits Lung Cancer Cells Proliferation

**Figure 2:**
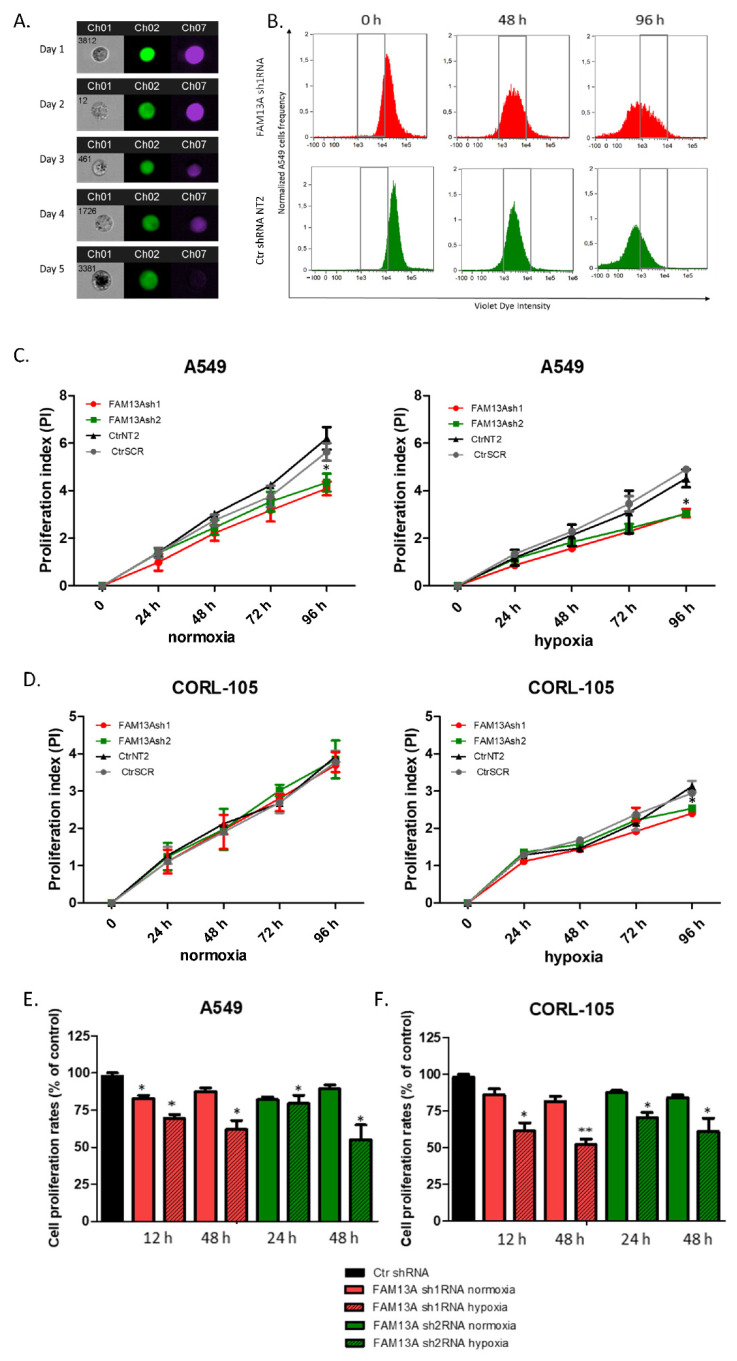
Silencing of FAM13A decreases lung cancer cell proliferation. Decreased A549 and CORL-105 cell proliferation after *FAM13A* gene knockdown was assessed using Violet Proliferation Dye (VPD450) and MTS test. After VPD450 staining the cells were cultured for 4–5 days in hypoxia and normal oxygen tension. The fluorescence intensity was measure by flow cytometry. During MTS test the cells were cultured in 96-well plates and cultured for two days in normal and hypoxic atmosphere. After 24/48 h the CellTiter 96^®^ AQueous One Solution Reagent was added and the absorbance was read at 490 nm with a microplate reader. (**A**) Representative images of A549 cells stained with VPD450. Fluorescence intensity was measured from Day 1 (0 time point) to 5 (96 h time point) by flow cytometry (Flow Sight^®^, Amnis, Seattle, WA, USA). Each cell is represented by a row of three images acquired simultaneously in flow, from left to right: channel 1—brightfield (gray), channel 2—fluorescence from GFP (transduced cells green) and channel 7—fluorescence from dye VPD450 applied to viable cells (purple). (**B**) Representative histogram plots displaying changes in VPD450 fluorescence intensity of A549 cells upon FAM13A knockdown (FAM13Ash1, red) and control (CtrNT2, green), in three time points: 0 h, 48 h, 96 h. (**C**,**D**) Cell growth kinetics was determined by proliferation index (PI) of FAM13Ash1 (red), FAM13Ash2 (green) and CtrNT2 (black), CtrSCR (grey) control cells in A549 (**C**) and CORL-105 (**D**). The difference in PI ratios at 24 h, 48 h, 72 h between FAM13A depleted cells and control cells was evaluated under normoxia (**left** panel) and hypoxia (**right** panel). A two-way ANOVA with Bonferroni posttest was used for statistical analysis. * *p* < 0.05 indicates a significant difference, as marked by an asterisk. The experiments were performed in triplicate and repeated three times. (**E**,**F**) Cell proliferation assessed by MTS assay. The results are given for A549 (**E**) and CORL-105 cells (**F**). Cell proliferation rate of FAM13Ash1 (red), FAM13Ash2 (green) cells and control cells (mean combined for CtrNT2 and SCR, black) was determined by CellTiter™ AQueous assay (MTS). The difference in cell proliferation rate of FAM13A depleted cells compared to control cells was evaluated under normoxia (bars without filling) or hypoxia (bars with texture) conditions. Data are means ± SEM (n = 4), * *p* < 0.05, ** *p* < 0.01, two-way ANOVA.

We performed a proliferation assay using two tests: Violet Proliferation Assay (VPD) and MTS. These assays measure cell proliferation indirectly. The first is based on labeling viable cells with cell tracking VPD450 dye (Figure 2A,B), and the second is based on the measurement of cellular metabolic activity. The results of VPD showed a significant reduction in A549 cells proliferation in FAM13Ash1 (PI = 3.05 ± 0.16) and FAM13Ash2 (PI = 3.73 ± 0.4) silenced cells compared to controls (CtrNT2 PI = 4.02 ± 0.71; CtrSCR PI = 4.41 ± 0.42), in hypoxia conditions (*p* < 0.05) (Figure 2C). Knockdown of *FAM13A* significantly reduced A549 cells proliferation also in normal oxygen tension, especially measured after 96 h (FAM13Ash1: PI = 4.09 ± 0.29; FAM13Ash2: PI = 4.34 ± 0.38; CtrNT2 PI = 5.95 ± 0.72; CtrSCR PI = 4.93 ± 0.34). In CORL-105 cells, FAM13A silencing significantly reduced cell proliferation (FAM13Ash1: PI = 2.4 ± 0.04; FAM13Ash2: PI = 2.54 ± 0.09; CtrNT2 PI = 3.13 ± 0.15; CtrSCR PI = 2.95 ± 0.02), after 96 h of hypoxic cultivation. It is worth mentioning that further incubation in hypoxia conditions, decreased CORL-105 proliferation rates (measured after 120 h) and had strong impact on cells viability (data not shown). The VPD assay showed that FAM13A silencing did not affect the CORL-105 cell viability in normoxia (Figure 2D). In summary, in both cell lines decreased cell proliferation under hypoxia conditions was observed.

The tendency of decreased proliferation in *FAM13A* depleted cancer cells was also observed in MTS assay (Figure 2E,F). Cell proliferation rates significantly decreased under hypoxia compared to normal oxygen tension (*p* < 0.05) in both A549 and CORL-105 cells (Figure 2E,F, respectively). Thus, we demonstrated that *FAM13A* silencing modulates the growth of cancer cells in hypoxia conditions.

To analyze the effects of FAM13A silencing on the cell cycle, BrdU assay followed by 7AAD staining was used to distinguish the percentage of the cellular population that is in each phase of the cell cycle (G1, S, and G2). A549 FAM13A depleted cell populations exhibited a slight decrease in the percentage of the cellular population in the S phase in normoxia compared to control cells (Figure S5). After hypoxia, the percentage of the cellular population in the S phase in A549 FAM13Ash1 and A549 FAM13Ash2 cells were significantly lower than observed for control cells (8.4% for FAM13Ash1/2 vs. 12.21% for controls; *p* < 0.05). This results revealed that FAM13A inhibition induced S phase cell cycle arrest in lung cancer tumor cells under hypoxia conditions.

### 2.3. Knockdown of FAM13A Suppresses Lung Cancer Cells Migration

**Figure 3:**
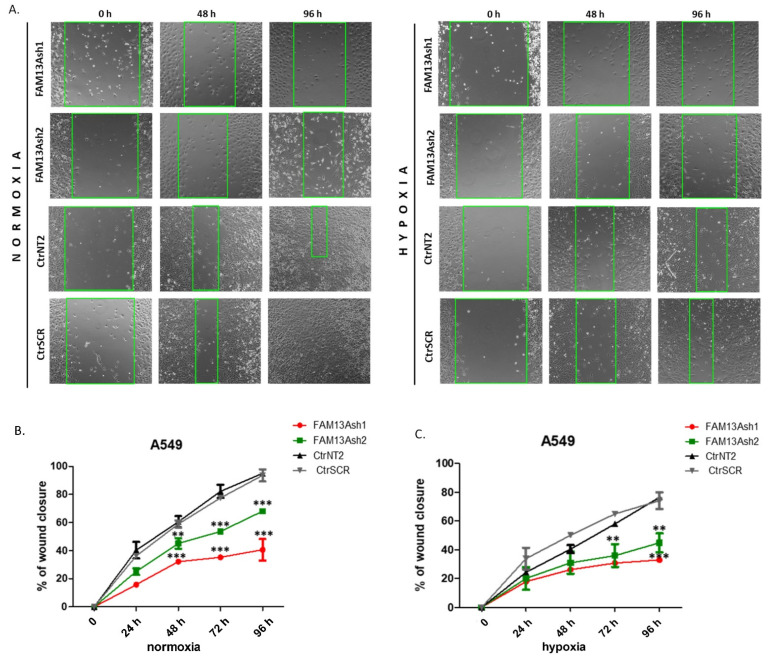
Knockdown of FAM13A suppresses A549 lung cancer cell migration. The migration of A549 cells with FAM13A knockdown and controls was monitored for 96 h under normoxia and hypoxia conditions. The inserts were used to generate a defined gap for measuring the migratory rates. Cell suspension in low serum media was added to the open end at the top of the insert. The cells were incubated overnight to form the monolayer. After 24 h inserts were removed from wells. The wound healing closure was visualized under a light microscope, images were taken in 24 h intervals. (**A**) Representative microscopy images of the wound-healing assay (magnification, ×10). Images of FAM13A depleted cells (FAM13Ash1) and control (CtrSCR) cells, cultured under normoxia (**left** panel) and hypoxia (**right** panel), were taken at the time 0 h, 48 h and 96 h after wounding. (**B**,**C**) Wound confluence (% of wound closure) was measured at 5 time points (0 h, 24 h, 48 h, 72 h, 96 h) after wound generation for FAM13A knockdown A549 cells: FAM13Ash1 (red), FAM13Ash2 (green) and controls: CtrNT2 (black), CtrSCR (grey) cultured in normal oxygen concentration (**B**) and under hypoxia (**C**). Data is expressed as the mean ± SEM of n = 3 exp. A two-way ANOVA with Bonferroni posttest was used for statistical analysis. *p* values ≤ 0.05 indicate a significant difference, as marked by an asterisk (** *p* < 0.01, *** *p* < 0.001). The experiments were performed in triplicate and repeated three times.

**Figure 4:**
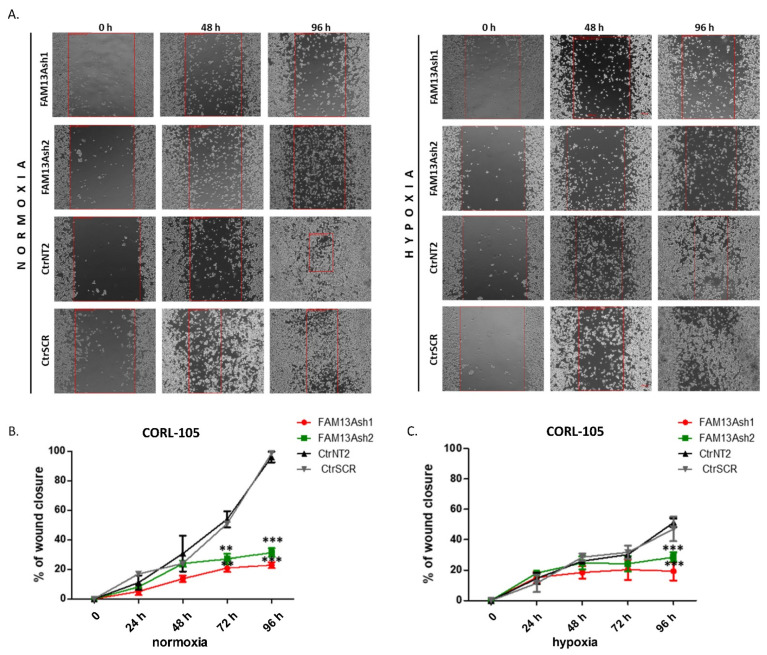
Knockdown of FAM13A suppresses CORL-105 lung cancer cell migration. The migration of A549 cells with FAM13A knockdown and controls was monitored for 96 h under normoxia and hypoxia conditions. The inserts were used to generate a defined gap for measuring the migratory rates. Cell suspension in low serum media was added to the open end at the top of the insert. The cells were incubated overnight to form the monolayer. After 24 h inserts were removed from wells. The wound healing closure was visualized under a light microscope, images were taken in 24 h intervals. (**A**) Representative microscopy images of the wound-healing assay (magnification, ×10). Images of FAM13A depleted cells (FAM13A sh2RNA) and control (Ctr sh1RNA NT2) cells, cultured under normoxia (**left** panel) and hypoxia (**right** panel), were taken at the time 0 h, 48 h and 96 h of wounding. (**B**,**C**) Wound confluence (% of wound closure) was measured at 5 time points (0 h, 24 h, 48 h, 72 h, 96 h) after wound generation for FAM13A knockdown CORL-105 cells: FAM13A sh1RNA (red), FAM13A sh2RNA (green) and controls: Ctr shRNA NT2 (black), Ctr shRNA SCR (grey) cultured in normal oxygen concentration (**B**) and under hypoxia (**C**). Data is expressed as the mean ± SEM of n = 3 exp. A two-way ANOVA with Bonferroni posttest was used for statistical analysis. *p* values ≤ 0.05 indicate a significant difference, as marked by an asterisk (** *p* < 0.01, *** *p* < 0.001). The experiments were performed in triplicate and repeated three times.

To elucidate the influence of *FAM13A* gene on lung cancer cells migration we applied the wound healing assay. Figure 3 and Figure 4 show representative images taken during A549 and CORL-105 cells monitoring. The wound healing assay revealed that the closing rate of scratch wounds was significantly decreased in hypoxia compared to cells cultured under normal oxygen conditions in both cell lines (*p* < 0.05, Figure 3 and Figure 4). The percentage of wound closure (WC) for control cells in normal atmospheric conditions reached 95–100%, whereas for cells exposed to 1% O_2_ for 96 h percentages were significantly decreased. The migration of A549 FAM13A sh1RNA and sh2RNA cells was significantly suppressed in both oxygen tensions, especially after 72 and 96 h (*p* < 0.001 in normoxia, *p* < 0.01 after hypoxia). However, stronger reduction in cell migration to the wound area was observed in normoxia (Figure 3B). All results obtained for A549 cells in normal oxygen concentration were confirmed using wound healing monitoring system (JuLI FL) (Figure S2).

More severe effect on inhibition of cell migration was observed for CORL-105 FAM13A depleted cells exposed to 21% O_2_ condition (Figure 4B,C). Together, the wound healing results showed that FAM13A silencing has a strong negative impact on cell migration, which was more pronounced in CORL-105 cells.

### 2.4. FAM13A Knockdown Inhibits Invasion of Lung Cancer Cells

**Figure 5:**
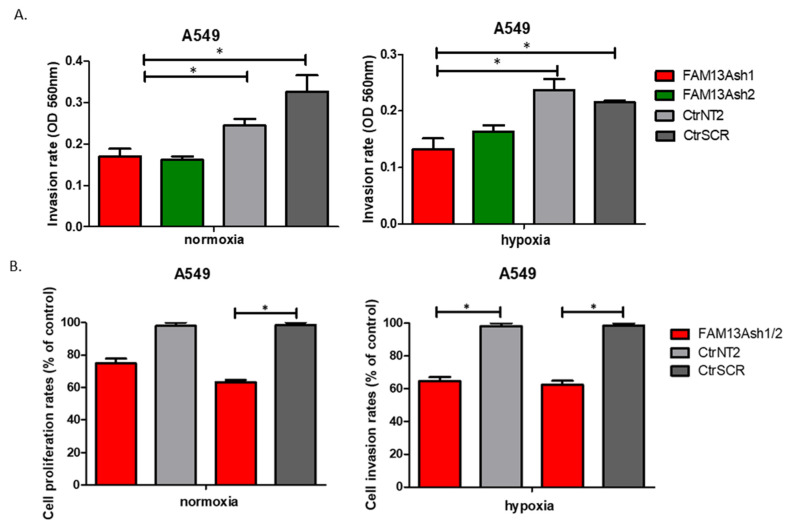
FAM13A knockdown inhibits invasion of A549 lung cancer cells. The influence of FAM13A silencing on the invasive properties of A549 cells was analyzed by the transwell invasion assay. Amount of A549 invasive cells were quantified using a colorimetric method (OD 560 nm) after 48 h of culture in normoxia and hypoxia. (**A**) OD values of invasive A549 FAM13Ash1 (red), FAM13Ash2 (green) cells were decreased remarkably compared with control CtrNT2 (light grey), CtrSCR (dark grey) cells, cultured under normoxia (**left** panel) and hypoxia (**right** panel). Data is expressed as the mean ± SEM of n = 3 exp., student’s *t*-test (two-tailed), * *p* < 0.05. (**B**) Averages of invasion rates are presented for A549 FAM13Ash1 and FAM13Ash2 (red), cells measured as percent of control CtrNT2 (light grey), CtrSCR (dark grey) cells, cultured under normoxia (**left** panel) and hypoxia (**right** panel). Data is expressed as the mean ± SEM of n = 3 exp., student’s *t*-test (two-tailed), * *p* < 0.05.

Boyden chamber invasion assay was used to analyze the influence of FAM13A silencing on the invasive properties of the two lung cancer cell lines. Cell invasion rates were significantly decreased in FAM13A depleted A549 cells compared to controls (Figure 5). OD values of invasive A549 FAM13Ash1, FAM13Ash2 cells were reduced compared with CtrNT2, CtrSCR control cells, cultured under normoxia and hypoxia. The most significant decrease in A549 cell invasion rate (%) was observed for FAM13Ash2 cells vs. CtrNT2 (M = 73.93 ± 8.1) in normoxia and FAM13Ash1 cells vs. CtrNT2 (M = 54.2 ± 2.0), FAM13Ash1 cells vs. CtrSCR (M = 49.2 ± 2.5) under hypoxia. The same experiment was conducted in CORL-105 cells, however we did not observe any significant effect on invasive phenotype of cells with *FAM13A* knockdown (data not shown). Based on these results, we concluded that FAM13A shRNA inhibits invasion of lung cancer in a cell type-specific manner.

### 2.5. FAM13A Silencing Promotes Changes in the Cytoskeleton

**Figure 6:**
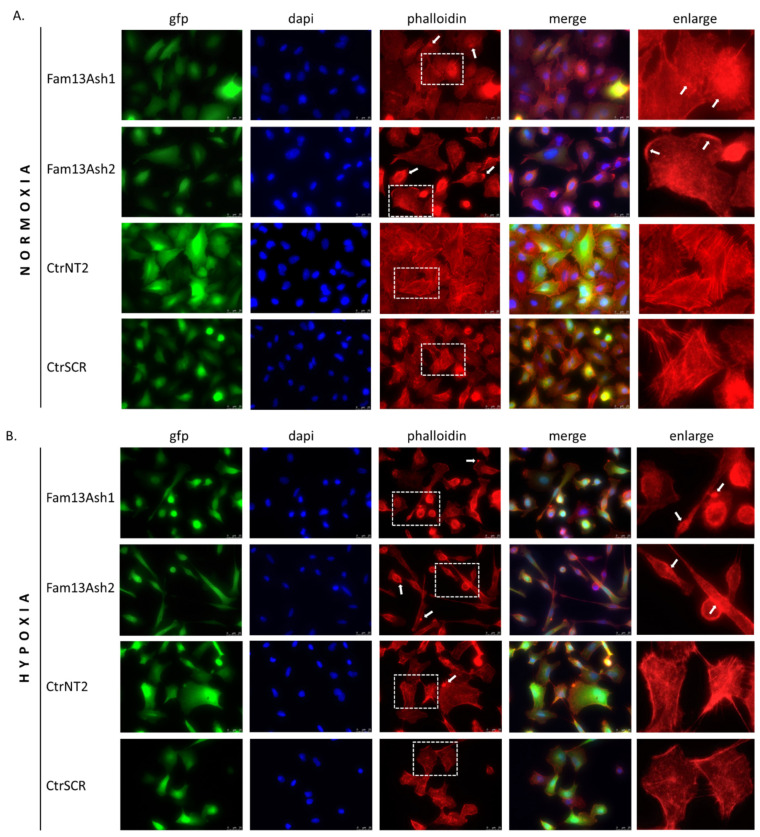
Stable knockdown of FAM13A promotes F-actin cytoskeletal reorganization. The FAM13A depleted cells (FAM13Ash1, FAM13Ash2) and control (CtrSCR, CtrNT2) cells, were cultured under normoxia and hypoxia for 72 h. After incubation, the cells were fixed, permeabilized and stained with Alexa Fluor^®^ 568 phalloidin for actin filaments visualization and DAPI for nuclei. The cells were observed under a fluorescence microscope. Example images (scale bar 25 μm) of the diverse F-actin organization phenotypes induced by FAM13A gene silencing in normoxia (**A**) and chronic hypoxia (**B**). A549 cells with stable FAM13A knockdown (gfp positive) were stained with phalloidin for F-actin (red), with DAPI for nuclei (blue). Representative single and overlaid images of immunofluorescence staining were present (magnification, ×63). The arrowheads indicate F-actin aggregates and accumulations of F-actin punctae.

As the cell migration depends on the actin cytoskeleton we assessed the influence of FAM13A inhibition on the cytoskeleton phenotype. By using phalloidin to dye fibrous actin (F-actin), we observed several changes in A549 cells cytoskeleton (Figure 6). We showed that FAM13A inhibition resulted in changes in cell shape, loss of cell-cell contacts, and sparsely located cells especially after chronic hypoxia (Figure 6B). Disruption of the actin cytoskeleton were visualized by the reorganization of F-actin filaments and presence of the rounding-up of cells, the large F-actin aggregates and the formation of numerous F-actin punctae. The observed cytoskeleton changes were present among A549 FAM13A depleted cells in normal oxygen tension (Figure 6A) and was strongly enhanced after hypoxia (Figure 6B). These results demonstrated that the loss of FAM13A in A549 cells led to cytoskeletal disruptions and inhibit cell migration.

### 2.6. Silencing of FAM13A Has No Effect on Apoptosis

The VPD and Annexin-V/7AAD staining showed that FAM13A silencing had no effect on apoptosis induction in A549 and CORL-105 cells. We did not observe increase in percentage of cells undergoing apoptosis and there were no significant differences between FAM13A depleted A549/CORL-105 cells compared to controls (CtrNT2, CtrSCR), neither in normal O_2_ tension, nor in hypoxia conditions (Figure S3).

## 3. Discussion

The function of FAM13A in non-small cell lung cancer (NSCLC) progression under hypoxic condition is unknown. In the present study, we focused on the role of FAM13A in cellular processes such as proliferation, cell cycle, migration, invasion and apoptosis in NSCLC cells under hypoxia. All these features are relevant for tumor progression and manifestation of metastases. To this end we used A549 and CORL-105 cells with stable FAM13A knockdown.

In our previous report, induction of FAM13A in lung cancer cells was confirmed in both in vitro and ex vivo models in short-term cultures after 72 h of hypoxia [6]. In the current study, significant increase in FAM13A expression was confirmed after exposure to chronic hypoxia in both cell lines (Figure 1). So far, few data are available on the FAM13A hypoxia-dependent regulation and its contribution to lung cancer progression. Studies on gene expression signature of the hypoxia response derived from several tissues (not comprising the lung) have shown a consistent increase of FAM13A expression [15].

In the literature there is only one study focused on the expression and function of FAM13A in NSCLC cells [14]. Among patients with lung adenocarcinoma and squamous cell carcinoma, increased FAM13A levels were found in tumoral area of the lung. Furthermore, FAM13A mRNA level together with HIF1α subunit were increased in antitumor CD4 + CD25 T effector cells in the hypoxic lung region of NSCLC patients. It was hypothesized that FAM13A together with HIF1α might be associated with antitumor effector T cells in the control area surrounding the growing tumor, in which oxygen deprivation occurred [14].

Recently, Godet et al. identified gene expression patterns in breast cancer cells that experienced intratumoral hypoxia in vivo compared to cells exposed to deoxygenation in vitro, with the use of unique hypoxia fate-mapping system. Set of 19 genes, including FAM13A, was induced by hypoxia in primary tumor and remained overexpressed at metastatic site in the lung, suggesting occurrence of a ‘hypoxic memory’. It was suggested that hypoxia inducible genes can be used as biomarkers to identify cells at metastatic sites that have been exposed to intratumoral hypoxia [16]. We can assume that *FAM13A* gene is a crucial member of hypoxia-response gene set in NSCLC, however our understanding of its contribution in lung cancer progression is still limited.

To examine the impact of *FAM13A* gene silencing on lung cancer cells proliferation under hypoxia, we performed series of tests, including VPD assay, MTS test and also BrdU assay. The results of VPD assay show a significant reduction in cell proliferation after FAM13A knockdown in A549 cells cultured in normal oxygen tension, as well as hypoxia, while the effect of FAM13A downregulation on CORL-105 cells was observed only after 96 h exposition to hypoxia. It is worth mentioning that hypoxia decreased cell proliferation in both cell lines, regardless of FAM13A knockdown. We also observed that oxygen deprivation had a stronger impact on CORL-105 cells growth (data not shown), compared to A549 cells. In MTS assay, negative effect of FAM13A shRNA on A549 and CORL-105 cell proliferation was observed already after 24 h and 48 h, especially under hypoxia conditions. The observed differences between VPD450 and MTS assays is probably due to their underlying methodology. In the current study, MTS method appeared to be more sensitive for cell proliferation assessment. Moreover, it was confirmed that VPD450 tracking of cancer cell division could be difficult [17]. Cancer cells are less homogeneous in size and protein content and consequently show broader peaks in histograms, which makes the quantitative population analyses more difficult [17]. We also observed this phenomenon during experiments. The third method used for proliferation assessment in the current study (BrdU assay) confirmed that FAM13A inhibition induced S phase cell cycle arrest in lung cancer tumor cells under hypoxia conditions. To summarize, very little is known about the role of *FAM13A* gene on tumor cellular proliferation. Only in one study, decrease in A549 cell number and proliferation was observed after FAM13AsiRNA treatment [14]. In line with this, our results confirmed that FAM13A drives NSCLC cell proliferation, especially under hypoxia.

To analyze the role of FAM13A in cancer cell metastasis, we performed wound healing and invasion assays. The wound healing results showed that FAM13A silencing had a strong negative impact on A549 and CORL-105 cell migration (Figure 3 and Figure 4). We observed that while A549 cells responded faster to hypoxia (48 h), CORL-105 cells were overall more sensitive to hypoxia, which was reflected in more severe inhibition of migratory capacities after 72 and 96 h. In the current study we observed that hypoxia reduced cell migration, and FAM13A silencing resulted in further inhibition of cell migration both in normoxia and hypoxia.

As the cell migration depends on the actin cytoskeleton we assessed the influence of FAM13A inhibition on the cytoskeleton phenotype. The phalloidin stanning revealed that FAM13A depleted cells beside different cell shapes were characterized by various actin cytoskeleton disturbances. The reorganization of F-actin filaments and presence of F-actin aggregates were observed in normoxia and enhanced in hypoxia. The structural dysregulation of the actin cytoskeleton correlate with movement of tumor cells. Based on current results we assumed that FAM13A is involved in inhibition of cell migration via altering the actin cytoskeleton.

HIFs are induced rapidly upon exposure to 1% O_2_ and the delayed effect of hypoxia on migration may reflect the time required to inhibit a downstream signaling pathway in which FAM13A may be involved. However, the results of current study are opposite to reported by Eisenhut et al. in which transient knockdown of *FAM13A* in A549 cells caused induction of cell migration, probably due to Rho proteins activation [14]. On the other hand, the proliferation of FAM13A siRNA treatment cells was decreased, which was confirmed in this study. Some discrepancies may be due to the experimental setups. In our study we generated stable FAM13A knockdown in lung cancer cells, which allowed us to conduct experiments during longer time period. Moreover, during the migration assay, the wound was generated using inserts with a defined gap, which increases the accuracy and reproducibility of the experiments [18]. We also used cell starvation procedure before starting the experiments. Results obtained for A549 cells were confirmed by monitoring on JuLIFL system for live cells imaging.

In addition, we showed that FAM13A knockdown inhibits the invasive activities of NSCLC cells. Significant decrease in cell invasion rate was observed for FAM13A shRNA A549 cells vs. controls, under both oxygen concentrations (Figure 5). The inhibition of invasive properties of A549 FAM13A depleted cells was enhanced under hypoxia as compared with normoxia. The migration and invasion of FAM13A A549 depleted cells depleted may be linked to the epithelial-mesenchymal transition (EMT). It was shown that hypoxia may induce a partial EMT phenotype in NSCLC cell lines [19]. Moreover, recent data indicated that FAM13A may regulate the Wnt signaling activity associated with EMT in A549 cells [20]. Based on the current results we assume that the hypoxia-induced FAM13A overexpression may diminish the occurrence of EMT in lung cancer cells, but further studies are needed to verify this hypothesis.

The difference in effect of FAM13A knockdown on cellular processes between A549 and CORL-105 cells may be related to the cells of origin. A549 cell line was derived from primary lung carcinoma, whereas CORL-105 originated from metastasized lung adenocarcinoma (pleural effusion). We chose two different NSCLC cell lines to examine the effect of FAM13A suppression on cells from primary and metastatic site. It is possible that hypoxia promotes metastasis by modulating changes inherent only to cells in primary tumor. It was shown that cells exposed to hypoxia in a primary tumor have four-fold greater probability of becoming viable circulating tumor cell. Moreover, post-hypoxic cells have ability to form lung metastases, suggesting that they have an enhanced metastasis-initiating capability [14]. If FAM13A overexpression plays an adaptative role to hypoxia in cancer cells, silencing of this gene should have more severe effect on features of primary tumor cells. Our results confirmed this hypothesis, because FAM13A silencing caused milder inhibition of the growth and proliferation in cells derived from metastatic site. Moreover, FAM13A silencing had no influence on the invasive properties of CORL-105 cells.

In the current study, we focused on downregulation of functional isoform 1 of FAM13A protein (117 kDa), which contains Rho GTPase activating protein (GAP) domain. It was reported that the reduced expression of FAM13A resulted in increased RhoA activation in A549 cells and primary human bronchial epithelial cells from cystic fibrosis patients [13]. RhoA protein function during cell migration and invasion was confirmed although this contribution depended on the environment and cell type [21]. In our study, we demonstrated that FAM13A knockdown inhibits the invasive activities and migration of NSCLC cells, which may be connected with the Rho GTPase pathways and also with hypoxia induced pathways. The effect of FAM13A inhibition on cell migration/invasion can be related to great plasticity of cancer cells in their movement mechanisms [22] and to the activation of other signaling pathways involved in cell migration, e.g., Rac GTPase-mediated signaling [23]. However, the detailed mechanism of this process needs to be verified.

Numerous studies demonstrated that the Rho/Rho-kinase signaling pathway may be involved in the apoptosis of lung cancer cells [24]. Thus, we decided to investigate if silencing of FAM13A gene expression affects the apoptosis in NSCLC cells. We found that FAM13A knockdown did not induce apoptosis in A549 or CORL-105 cells. Previously, it was shown that TGFβ induced apoptosis in A549 cells and it was associated with downregulation of two main FAM13A isoforms [14]. Further studies are necessary to better understand the role of FAM13A in programmed tumor cell death.

In summary, we confirmed that FAM13A silencing has a negative effect on proliferation, migration via altering the actin cytoskeleton, and invasion activity in NSCLC cells in normal and hypoxic conditions. Our study showed that oxygen deprivation further enhanced phenotypic changes in FAM13A depleted cells. We demonstrated that FAM13A depleted post-hypoxic cells have a decreased metastatic potential, which indicates FAM13A as a potential therapeutic target in lung cancer.

## 4. Materials and Methods

### 4.1. Materials

A549 (human non-small cell lung carcinoma) cell line was purchased from ATCC^®^, CORL-105 (Caucasian lung adenocarcinoma) cell line was purchased from ECACC^®^ (The European Collection of Cell Cultures), supplied by Sigma-Aldrich (St. Louis, MO, USA), and lentivirus producer cell line (HEK-293T) was purchased from DSMZ. Cells were cultured in DMEM (Sigma Aldrich) (A549 and HEK293T) or RPMI 1640 (Lonza, Basel, Switzerland) (CORL-105) supplemented with 10% fetal bovine serum (FBS, Sigma-Aldrich), 1% penicillin/streptomycin, 4 mM L-glutamine in a humidified atmosphere with 5% CO_2_ at 37 °C. Cell lines were cultured according to ATCC/ECACC/DSMZ protocols. Cells were maintained in a humidified atmosphere with 5% CO_2_ and 95% air (normoxia) at 37 °C. For hypoxic conditions, cells were incubated in a hypoxic incubator (BINDER, CB53) with a humidified atmosphere of 5% CO_2_ and 1% O_2_ balanced with N_2_ (hypoxia). Exponentially growing cells were used throughout the study. All cell cultures were regularly checked for mycoplasma contamination by PCR and proved negative.

### 4.2. Plasmids and Constructs

Short hairpin RNAs (shRNAs) were designed using the UCSC Broad Institute GPP Web Portal (Figure 1A) [25]. Two independent shRNAs against FAM13A transcripts targeting different regions were chosen. shRNAs were designed against a coding sequence and targeted the majority of FAM13A isoforms containing the RhoGAP functional domain, essential for the protein function. shRNA template oligonucleotides were synthesized (Genomed, Poland)—FAM13A sh1 sense: 5′-GATCCGCAAGCCTAAACGTCAGAAATTTCAAGAGAATTTCTGACGTTTAGGCTTGCTTTTTG-3′; FAM13A sh1 antisense: 5′-AATTCAAAAAGCAAGCCTAAACGTCAGAAATTCTCTTGAAATTTCTGACGTTTAGGCTTGCG-3′; FAM13A sh2 sense: 5′-GATCCGGACAAATGACCTTGCCAAATTTCAAGAGAATTTGGCAAGGTCATTTGTCCTTTTTG-3′; FAM13A sh2 antisense: 5′-AATTCAAAAAGGACAAATGACCTTGCCAAATTCTCTTGAAATTTGGCAAGGTCATTTGTCCG-3′—annealed and cloned into a pGreenPuro lentiviral vector (System Biosciences, Palo Alto, CA, USA) backbone cut with EcoRI and BamHI (Thermo Scientific, Waltham, MA, USA). Annealed oligonucleotides were ligated into vector using T4 ligase. Two non-targeting control shRNA constructs were used: NT2—NT2 sense: 5′-GATCCGCAACAAGATGAAGAGCACCAACTCTTCAAGAGAGTTGTTCTACTTCTCGTGGTTGAGTTTTTG-3′; NT2 antisense: 5′-GCGTTGTTCTACTTCTCGTGGTTGAGAAGTTCTCTCAACAAGATGAAGAGCACCAACTCAAAAACTTAA-3′—and scrambled (SCR, obtained from System Biosciences). Control NT2 and SCR vectors were a kind gift from prof. Anke van den Berg and Dr. Joost Kluiver [26]. All constructs were verified by Sanger sequencing. Detailed cloning information can be provided upon request.

### 4.3. Lentiviral shRNA Transduction

3rd generation 4-plasmid system for lentiviral particles production was used. Lentiviral vectors encoding FAM13Ash1, FAM13Ash2, CtrNT2 or CtrSCR were co-transfected with packaging vectors pMSCV-VSV-G, pRSV.REV and pMDL-gPRRE into HEK-293T cells using Calcium Phosphate Transfection Kit (Invitrogen, Carlsbad, CA, USA). Lentiviruses were generated according to the manufacturer’s protocol. Briefly, supernatants containing lentiviruses generated from HEK-293T cells were collected and filtered (0.45 µM syringe filter) 48 h and 72 h post-transfection and stored at −80 °C. A549 and CORL-105 were seeded in a 6-well plate at a density of 2.5 × 10^5^ and 5 × 10^5^ cells/well, respectively. After 24 h virus supernatant was added to cells together with polybrene (4 ug/mL). Medium was replaced after 24 h. The transduction efficiency was determined by flow cytometry (FlowSight, Amnis, Seattle, WA, USA) and fluorescence microscopy (Axio Vert A1, Zeiss, Oberkochen, Germany) based on percentage of GFP positive cells. The infection efficiency was over 90% in A549 cell line. For CORL-105 cells selection with 1.5 µg/mL puromycin was performed 48 h after transduction and the percentage of GFP-positive cells after 96 h of culture was over 90%. The names of generated cell lines were as follows: for FAM13A knockdown cells FAM13Ash1, FAM13Ash2 (cells transduced with FAM13A sh1RNA and sh2RNA vector); for control cells CtrNT2, CtrSCR (cells transduced with control NT2 and SCR vector).

### 4.4. Quantitative Analysis of mRNA Expression

A549 and CORL-105 cells were collected 7 days after transduction and washed with cold phosphate-buffered saline (PBS) twice. Then, 600 µL of cell lysis buffer RLT with β-mercaptoethanol (β-ME) was added to cell pellet. Total RNA was extracted using the RNeasy Mini Kit (Qiagen, Los Angeles, CA, USA) according to the manufacturer’s protocol and then 500 ng of RNA was reverse transcribed into cDNA using the QuantiTect Reverse Transcription Kit (Qiagen, USA). Quantitative real-time PCR analysis was performed using TaqMan probes directed at FAM13A (Thermo Fisher, Hs00208453_m1) and GUSB (Thermo Fisher, Hs00939627_m1) as a reference gene. The reactions were performed with an aliquot of cDNA equivalent of 5 ng total RNA and HOT FIREPol Probe qPCR Mix Plus (no ROX) according to the manufacturer’s instructions (Solis Biodyne, Tartu, Estonia) and under conditions specified in the TaqMan Gene Quantification assay protocol. Thermal cycling was performed using a CFX96 Touch™ Real-Time PCR Detection System (Bio-Rad, Hercules, CA, USA). The fold change value was calculated using 2^−ΔΔCt^, described by K. Livak et al. [27]. Each biological sample consisted of three technical replicates and three biological repeats were combined.

### 4.5. Western Blot

Cell lysis and protein extracts from A549 and CORL-105 cells were prepared using RIPA lysis buffer (R0278, Sigma) with addition of Protease Inhibitor Cocktail (PIC002.1, Bioshop, Canada). Protein concentration was determined using the Bicinchoninic Acid Kit, BCA (BCA1-1KT, Sigma). 30 µg of protein were mixed with Laemmli Sample Buffer (Sigma), resolved on the Mini-PROTEAN Stain-free gel (BioRad) with Mini-PROTEAN^®^ Tetra electrophoresis system (BioRad) and transferred to PVDF membranes using the Trans-Blot^®^ Turbo™ system. Membranes were blocked with 5% milk and incubated with anti-FAM13A antibody (ab122440, Abcam, 1:500, Cambridge, UK) overnight at 4 °C. After incubation with horseradish peroxidase-conjugated anti-rabbit secondary antibody (ab97051, Abcam, 1:50,000), the signal was detected by chemiluminescence with Clarity Western ECL Substrate using ChemiDoc™ Imaging Systems (BioRad). Normalization of FAM13A protein abundance was made in reference to total protein staining. Gel and blot images were analyzed by Image Lab™ Software (BioRad).

### 4.6. Cell Proliferation Assessment by MTS Test

Cell proliferation rate was determined by CellTiter™ AQueous assay (MTS, Promega, Madison, WI, USA). Cells were seeded at the following densities: A549: 5 × 10^3^ and 1 × 10^4^ cells/well and CORL-105: 1 × 10^4^ and 2 × 10^4^ cells/well, in 100 µL of standard medium in 96-well plates and cultured for two days in normal and hypoxic atmosphere. 20 µL of CellTiter 96^®^ AQueous One Solution Reagent ([3-(4,5-dimethylthiazol-2-yl)-5-(3-carboxymethoxyphenyl)-2-(4-sulfophenyl)-2H-tetrazolium, inner salt) was added to each well. After 4 h of incubation, the absorbance was read at 490 nm with a microplate reader (Bio Tek, ELx808, Winooski, VT, USA). Four technical replicates were prepared for each sample in three separate experiments. Cell proliferation rates were calculated by: (mean OD treated well [−blank])/(mean OD control well [−blank]) × 100, as described by Prabst et al. [28].

### 4.7. Violet Proliferation Dye

Violet Proliferation Dye 450 (VPD450, BD Horizon™, Piscataway, NJ, USA) was used for cell proliferation assessment. The violet laser excitable dye VPD450 emits maximally at 450 nm with minimal fluorescent spillover into the FITC channel, which is eligible for use with green fluorescent protein (GFP)-tagged cells. The optimal condition of VPD450 labeling were established for A549 (5 × 10^6^ cells/mL) and CORL-105 (1 × 10^6^ cells/mL). Cells were washed twice in 1 × PBS to remove any residual serum proteins and resuspended into a single cell suspension at a concentration of 1–10 × 10^6^/mL in 1 × PBS. Subsequently, 1 µL of 1 mM VPD450 stock solution (in DMSO) was added for each 1 mL of cell suspension for a final VPD450 concentration of 1 µM. After incubation in a 37 °C water bath for 20 min, cells were washed with 9 mL of 1 × PBS and centrifuged. The cell pellet was washed with 10 mL of complete medium with 10% FBS and centrifuged again. Cells were resuspended in complete medium (DMEM or RPMI1640, with 10% FBS) and cultured for 4–5 days in hypoxia and normal oxygen tension. Analysis by flow cytometry (Flow Sight^®^, Amnis) was taken every 24 h, in total 6 time points. Experiments were conducted in triplicates. Flow cytometry data were analyzed by IDEAS^®^ software (Image Data Exploration and Analysis Software, Amnis). Proliferation index (PI) was calculated with the formula: PI = Log[FInd/MFIall]/Log [2], with MFIall = median fluorescence intensity of all viable cells and FInd = peak fluorescence intensity of the viable non-divided cells, as described by Biburger [29]. The measurement of cell growth kinetics was done after proliferation index calculation (PI), where the VPD450 fluorescence intensity on the first day was used to set the “zero” population.

### 4.8. Cell Cycle Analysis

Additionally, for A549 cells a 5-bromo-2 deoxyuridine (BrdU) incorporation assay was conducted to monitor DNA replication using a APC-BrdU Flow Kit (BD Bioscience, San Jose, CA, USA) with 7AAD staining according to the manufacturer’s instructions. Cell data were collected with CytoFLEX S flow cytometer (Beckman Coulter).

### 4.9. Wound-Healing Assay (WH)

The wound-healing assay mimics tumor cell migration in vivo. The migration rates of the FAM13Ash A549 and CORL-105 versus control cells were evaluated using the wound healing assay under normoxic and hypoxic conditions. FAM13Ash A549/CORL-105 and control cells were incubated for 24 h in low serum concentration (serum starvation, 1% of FBS) to suppress cell proliferation. We used CytoSelect™ Wound Healing Assay Kit (Cell Biolabs, San Diego, CA, USA), which includes inserts that generate a defined gap of 0.9 mm for measuring the migratory rates. All experiments were performed according to the vendor protocol. The WH Inserts™ were placed in 24-well plate in the same direction. Cell suspension containing 0.25–0.5 × 10^6^ cells/mL in low serum media was added to the open end at the top of the insert. The cells were incubated overnight to form the monolayer. After 24 h inserts were removed from wells, cells were washed with media to remove dead cells and debris. Finally, fresh low serum medium was added to wells. Cells were incubated in normoxia and hypoxia for 96 h. The wound healing closure was visualized under a light microscope. Phase contrast images were taken in 24 h intervals. The migration rate was calculated as the percentage of area reduction or wound closure, as described by Rotzer et al. [30]. Each sample consisted of three technical and three biological repeats.

Additionally, A549 cells were monitored using the JuLI FL system (NanoEnTek, Korea) for live cells imaging, in normal oxygen tension. A549 cells were seeded in a 12-well plate at a density of 1–1.5 × 10^5^ cells/well and after 24 h of growth cells reached ~90% confluency as a monolayer. Then, the cells were cultured in DMEM with 1% FBS (low-serum culture media) for 24 h. After scratching, cells were cultured for additional 48 h in fresh low-serum culture media to allow wound healing. During culturing, images were taken at 6 h intervals and used to prepare the migration rate curves as well as the time-laps video. The video was prepared using the wound healing monitoring mode that is based on the analysis of cells confluency. The experiment was repeated three times.

### 4.10. Immunofluorescence

The A549 cells were grown on glass coverslips under hypoxia/normoxia for 72 h. The cells were washed and then fixed with 4% paraformaldehyde for 15 min. Fixed cells were permeabilized by treatment with 0.5% TritonX-100 for 15 min and blocked by incubation with 5% BSA in PBS for 1 h. The cells were then incubated with Alexa Fluor^®^ 568 phalloidin for 1 h, according to the manufacturer protocol. The cells were mounted in ProLong™ Gold Antifade Mountant with DAPI. The cells were observed under a fluorescence microscope, 63× magnification (Leica DMi8).

### 4.11. Cell Invasion Assay

The invasion assay was conducted using modified Boyden chamber by CytoSelect™ Cell Invasion Assay Kit (Cell Biolabs, USA). This assay uses polycarbonate membrane inserts (8 µm pore size). The upper surface of the insert membrane is coated with a basement membrane matrix solution. All experiments were done in accordance to the vendor protocol. The basement membrane layer of inserts was rehydrated by adding 300 µL of warm, serum-free media and incubation at room temperature for 1 h. Cell suspensions containing 0.4 × 10^6^ A549 cells/mL and 1.0 × 10^6^ CORL-105 cells/mL were prepared in serum free media (DMEM, RPMI1640, respectively, both containing 0.5% BSA). Overnight starvation was performed in both cell lines prior to running the assay. Then, 300 µL of cell suspension was added into the upper well and 500 µL of complete culture medium containing 10% of FBS was added into the lower well of the insert. After incubation for 48 h at 37 °C in 5% CO_2_, normal or hypoxic atmosphere, the medium was aspirated from the inside of the insert. In the upper chamber, non-invasive cells were removed with a cotton swab. Invasive cells, at the bottom of the insert, were placed in 200 µL of Extraction Solution and incubated for 10 min on an orbital shaker. Subsequently, 100 µL from each sample was transferred to a 96-well microtiter plate and the OD 560 nm was measured in a colorimetric plate reader (Bio Tek, ELx808). All experiments were conducted in three biological and two technical repeats.

### 4.12. Detection of Cell Apoptosis Using Flow Cytometry

APC Annexin V Apoptosis Detection Kit with 7-AAD (BD Biosciences, San Jose, CA, USA) was used for the identification of apoptotic and necrotic cells. All experiments were taken according to manufacturer’s protocol. A549 and CORL-105 cells were collected, washed twice with cold PBS and then resuspend in 1× Binding Buffer at a concentration of 1 × 10^6^ cells/mL. Subsequently, 1 × 10^5^ cells were stained with addition of 5 µL of APC Annexin V and 5 µL of 7-AAD. After incubation (15 min. at 25 °C, in the dark), 400 µL of 1× Binding Buffer was added to each tube and analyzed by flow cytometry within an hour (Flow Sight^®^, Amnis). In parallel, a set of control samples were used as follows:

1. unstained cells (wild type and after transduction with lentivectors with GFP marker),
2. apoptosis control: cells stained with APC Annexin V (cells that undergo apoptosis after addition of camptothecin, final concentration 6 µM, to 1 × 10^6^ A549 and CORL-105 wild type cells and incubation for 24 h at 37 °C),
3. necrosis control: cells stained with 7-AAD (A549, CORL-105 wild type cells incubated at 96 °C for 10 min.)

Experiments were prepared in triplicates. Analysis of flow cytometry results was performed using IDEAS^®^ (Image Data Exploration and Analysis Software, Amnis).

### 4.13. Statistical Analysis

All experiments were performed in triplicate if not mentioned otherwise. The results of multiple observations are presented as the mean ± SEM or as a representative result of more than three different separate experiments, unless otherwise stated. Data were analyzed with GraphPad Prism (GraphPad Prism 5 Software, San Diego, CA, USA), using the statistical test stated in the figure legends and values were considered significant at *p* < 0.05. Continuous data were compared using t-tests for two independent groups and one-way ANOVA for 3 or more groups. For evaluation of continuous outcomes over time, two-way ANOVA followed by Bonferroni’s post hoc test was used to analyze the time-course curve for the analyzed process (wound healing assay, proliferation index assessment by VPD450, MTS) [31].

## Data Availability

Data are available at request from the authors.

## Supplementary Materials

The following are available online at https://www.mdpi.com/article/10.3390/ijms22094302/s1, Figure S1. Generating lung cancer cell lines with FAM13A knockdown; Figure S2. Knockdown of FAM13A suppresses A549 lung cancer cell migration under normoxia; Figure S3. Silencing of FAM13A has no effect on apoptosis; Figure S4. Western blots for Figure 1; Figure S5. FAM13A depleted cells exhibit decreased proliferation.

## Abbreviations

CF	Cystic fibrosis
COPD	Chronic obstructive pulmonary disease
EMT	Epithelial-mesenchymal transition
FAM13Ash1	FAM13A sh1RNA vector
FAM13Ash2	FAM13A sh2RNA vector
HIFs	Hypoxia-inducible subunits
MTS	3-(4,5-dimethylthiazol-2-yl)-5-(3-carboxymethoxyphenyl)-2-(4-sulfophenyl)-2H-tetrazolium
NT2	non-targeting control shRNA construct
NSCLC	Non-small cell lung cancer
qRT-PCR	Quantitative Reverse Transcription Polymerase Chain Reaction
SCR	scrambled control shRNA construct
shRNAs	Short hairpin RNAs
VPD	Violet Proliferation Assay
WH	Wound-healing assay
