# Supplementary material for: Hypoxia-Induced FAM13A Regulates the Proliferation and Metastasis of Non-Small Cell Lung Cancer Cells
Source: Int J Mol Sci. 2021 Apr 21;22(9):4302. doi: 10.3390/ijms22094302 (PMC8122400; doi:10.3390/ijms22094302)
Supplement: Supplementary file 1 [file ijms-22-04302-s001.zip › ijms-1173017-supplementary.pdf]

Supplementary materials: Hypoxia-induced FAM13A regulates the proliferation and metastasis of non-small cell lung cancer cells. Iwona Ziółkowska-Suchanek, Marta Podralska, Magdalena Żurawek, Joanna Łaczmańska, Katarzyna Iżykowska, Agnieszka Dzikiewicz-Krawczyk, Natalia Rozwadowska

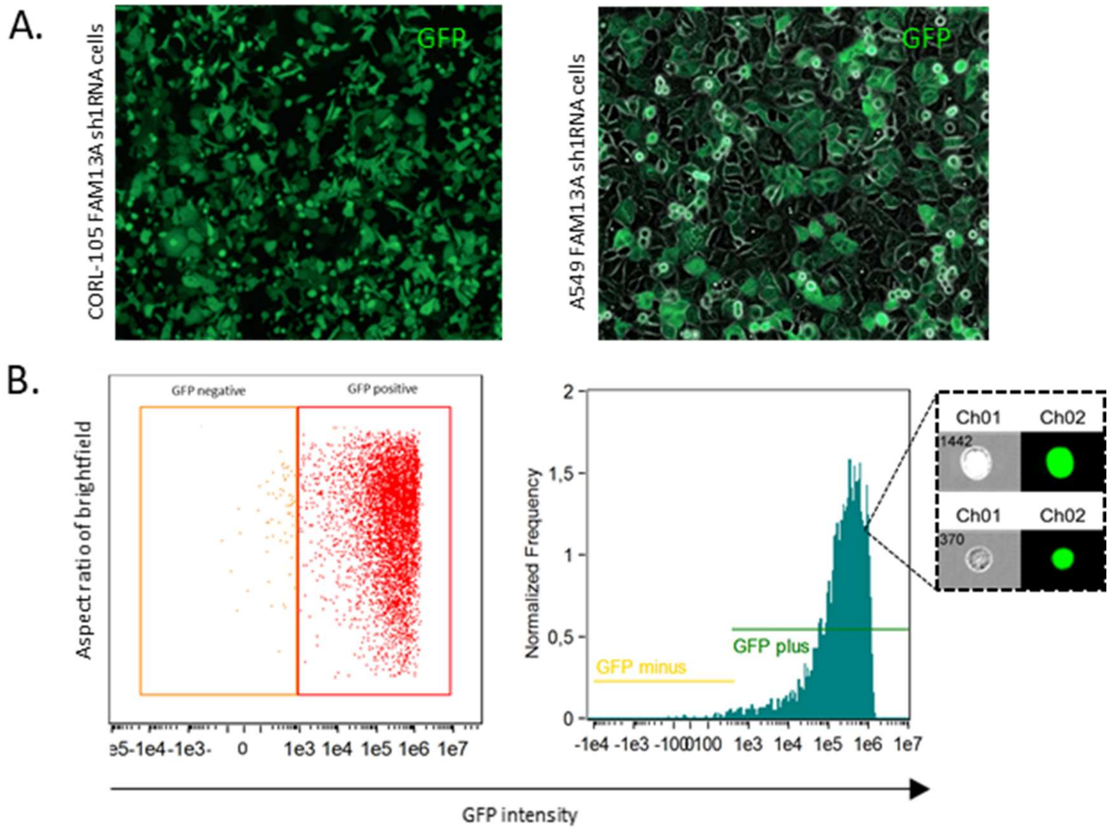

Figure S1. **Generating lung cancer cell lines with FAM13A knockdown.**
A. The transduction efficiency was determined by fluorescence microscopy (Axio Vert A1, Zeiss, Germany) based on percentage of GFP positive cells. Representative images of GPF positive cells were shown.
B. The transduction efficiency was determined by flow cytometry (FlowSight, Amnis, USA) based on percentage of GFP positive cells. Representative histograms of GFP positive cells were shown. The infection efficiency was over 90% in both cell lines.

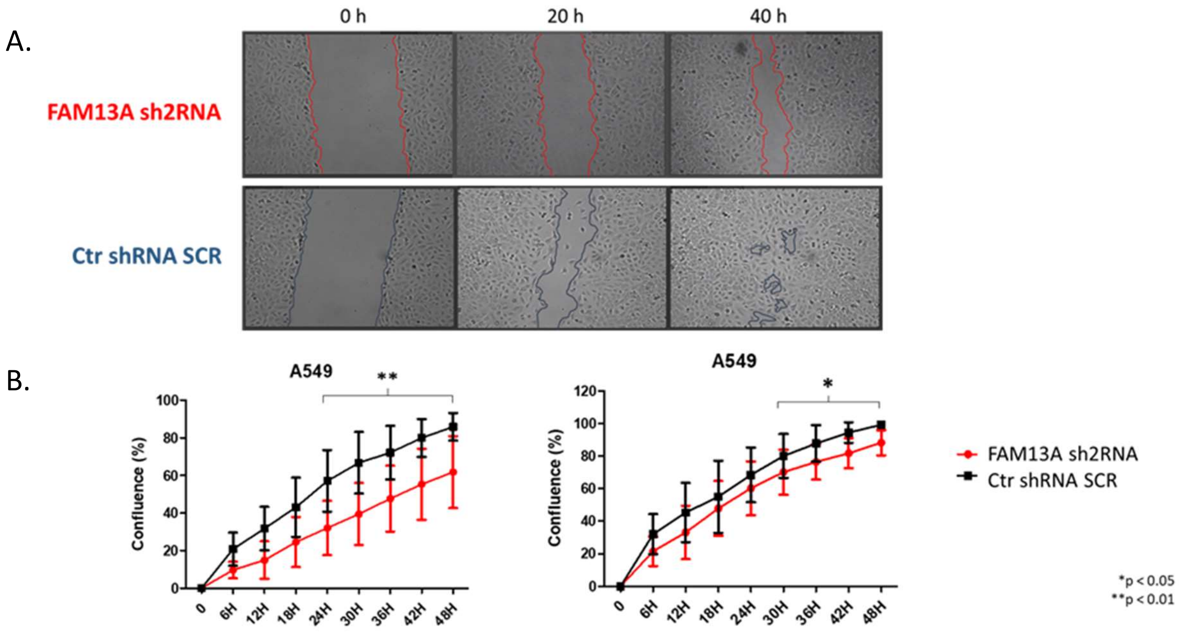

Figure S2. Knockdown of FAM13A suppresses A549 lung cancer cell migration under normoxia.
A. Representative images of the wound-healing assay. A549 cells were monitored with the use of JuLI FL system for live cells imaging, in normal oxygen tension. Images of FAM13A depleted cells (FAM13A sh2RNA) and control (Ctr shRNA SCR) cells, cultured under normoxia were taken at the time 0h, 20h and 40 hours of wounding.
B. Wound confluence (% of wound closure) was measured at 9 time points (0-48h) after wound generation for FAM13A knockdown A549 cells: FAM13A sh2RNA (red), Ctr shRNA SCR (black) cultured in normal oxygen concentration. Data is expressed as the mean ± SEM of n = 3 exp. A two-way ANOVA with Bonferroni posttest was used for statistical analysis. P values ≤ 0.05 indicate a significant difference, as marked by an asterisk (*p<0.05, **p < 0.01). The experiments were performed in triplicate and repeated three times.

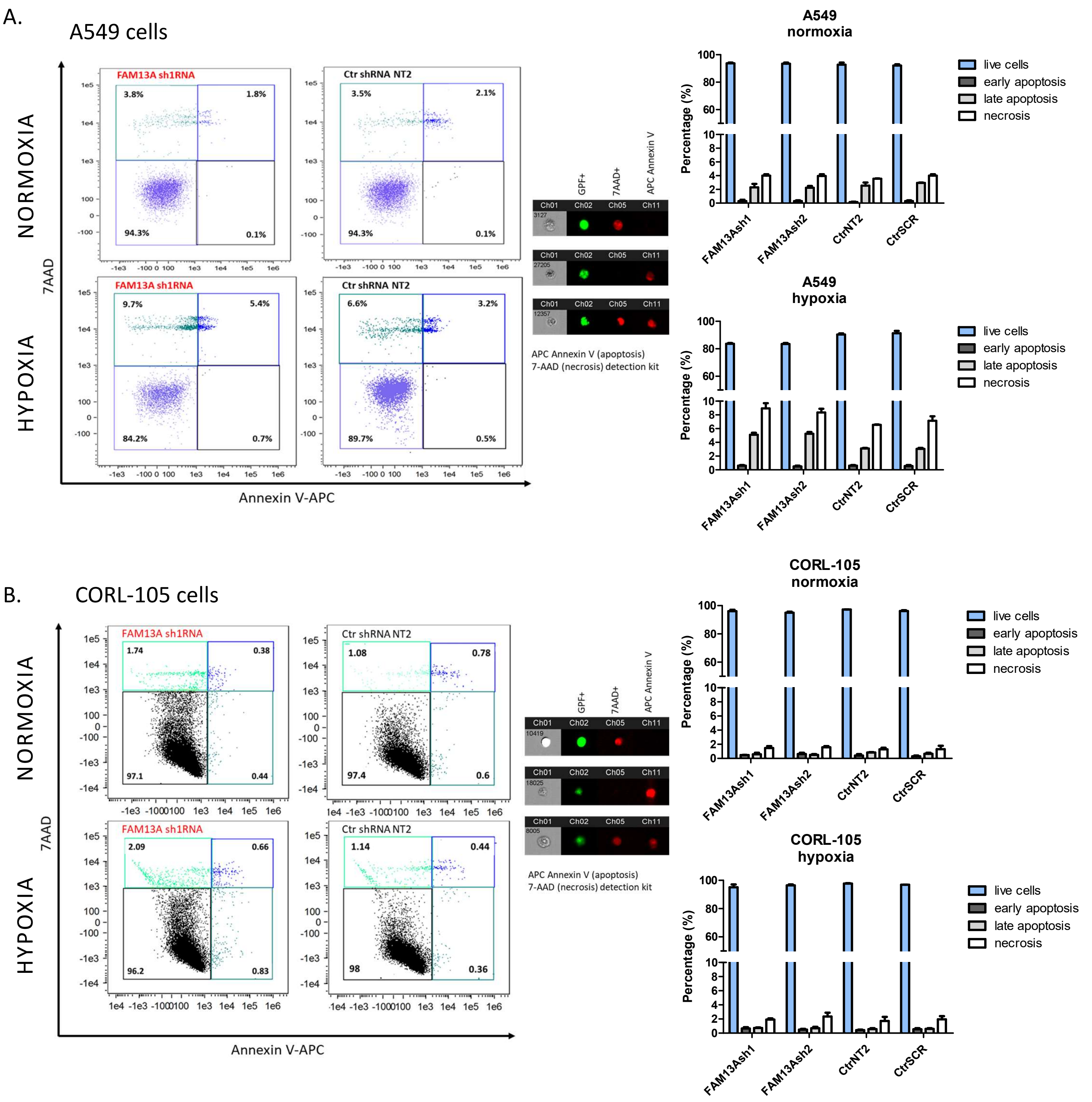

Figure S3. Silencing of FAM13A has no effect on apoptosis.
Flow cytometric analysis of apoptosis in A549 and CORL-105 cells after FAM13A knockdown. APC Annexin V Apoptosis Detection Kit with 7-AAD was used for the identification of apoptotic and necrotic cells cultured in normal and hypoxia conditions. Representative histograms are presented for FAM13Ash1RNA and control Ctr shRNA NT2 A549 (A) and CORL-105 (B) cells. Scatter plots representing the percentages of the of viable (APC−/7-AAD−), necrotic (APC−/7-AAD+), early apoptotic (APC+/7-AAD−) and late apoptotic (APC+/7-AAD+) cells. The bar charts present percentage of live cells, cells in early apoptosis, cells in late apoptosis and necrotic cells observed in both cell lines. Bars represent mean ± SEM of 3 independent experiments.

A.

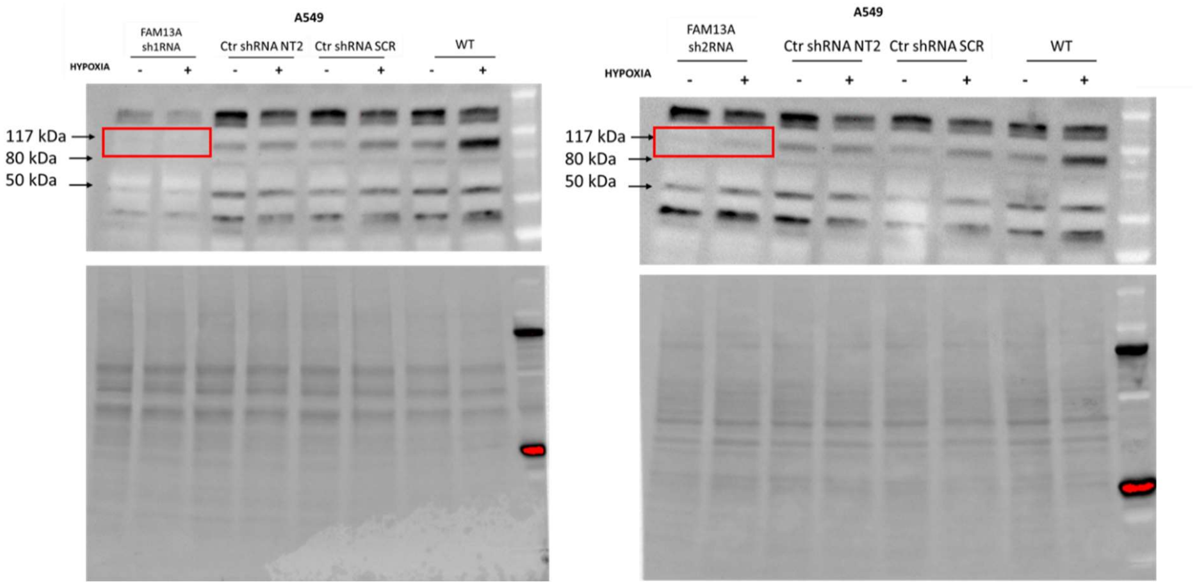
A549
FAM13A sh1RNA
Ctr shRNA NT2
Ctr shRNA SCR
WT
HYPOXIA
- + - + - + - +
117 kDa
80 kDa
50 kDa
A549
FAM13A sh2RNA
Ctr shRNA NT2
Ctr shRNA SCR
WT
HYPOXIA
- + - + - + - +
117 kDa
80 kDa
50 kDa

B.

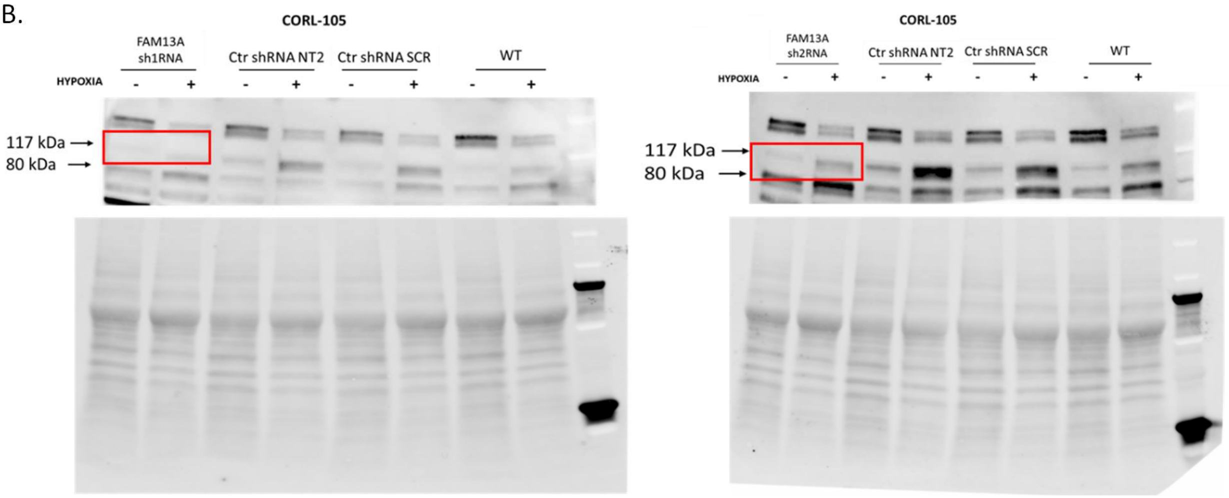
CORL-105
FAM13A sh1RNA
Ctr shRNA NT2
Ctr shRNA SCR
WT
HYPOXIA
- + - + - + - +
117 kDa
80 kDa
CORL-105
FAM13A sh2RNA
Ctr shRNA NT2
Ctr shRNA SCR
WT
HYPOXIA
- + - + - + - +
117 kDa
80 kDa

C.

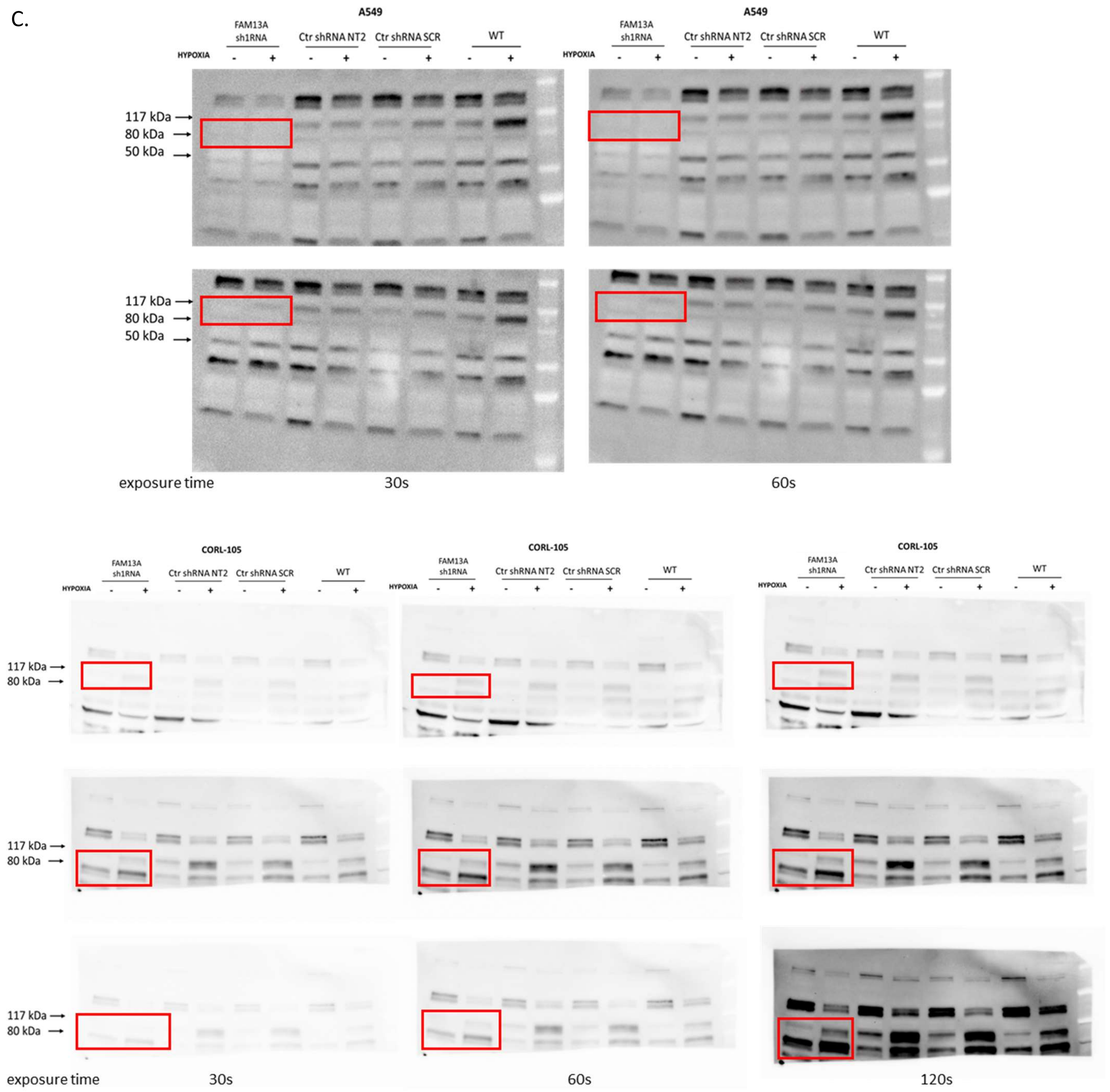

Figure S4. Western blots for Figure 1. Representative full-length blots (A-B, C) with different exposure time are presented (C). Membranes were often cut to enable blotting for multiple antibodies. Specific regions of the original blots cropped for the Figure 1 with FAM13A sh1/sh2RNA depleted cells are in red boxes.

A-B. Western blot (WB) analysis of FAM13A expression in A549 (A) and CORL-105 (B) cell lines cultured under normoxia and hypoxia for 72h. Relative FAM13A protein (117kDA) amount was significantly decreased in FAM13A sh1/sh2RNA cells. Strong FAM13A expression was induced in controls (Ctr shRNA NT2, Ctr shRNA SCR) and wild type (WT) A549 or CORL105 cells after 72h of hypoxia (+) compared to normaxia (-). The normalization of protein amount was made with Mini-PROTEAN Stain-free gel system (BioRad). Total protein was used for normalization. Representative stain-free total protein blots are presented.

C. Representative full-length blots with different exposure time are presented.

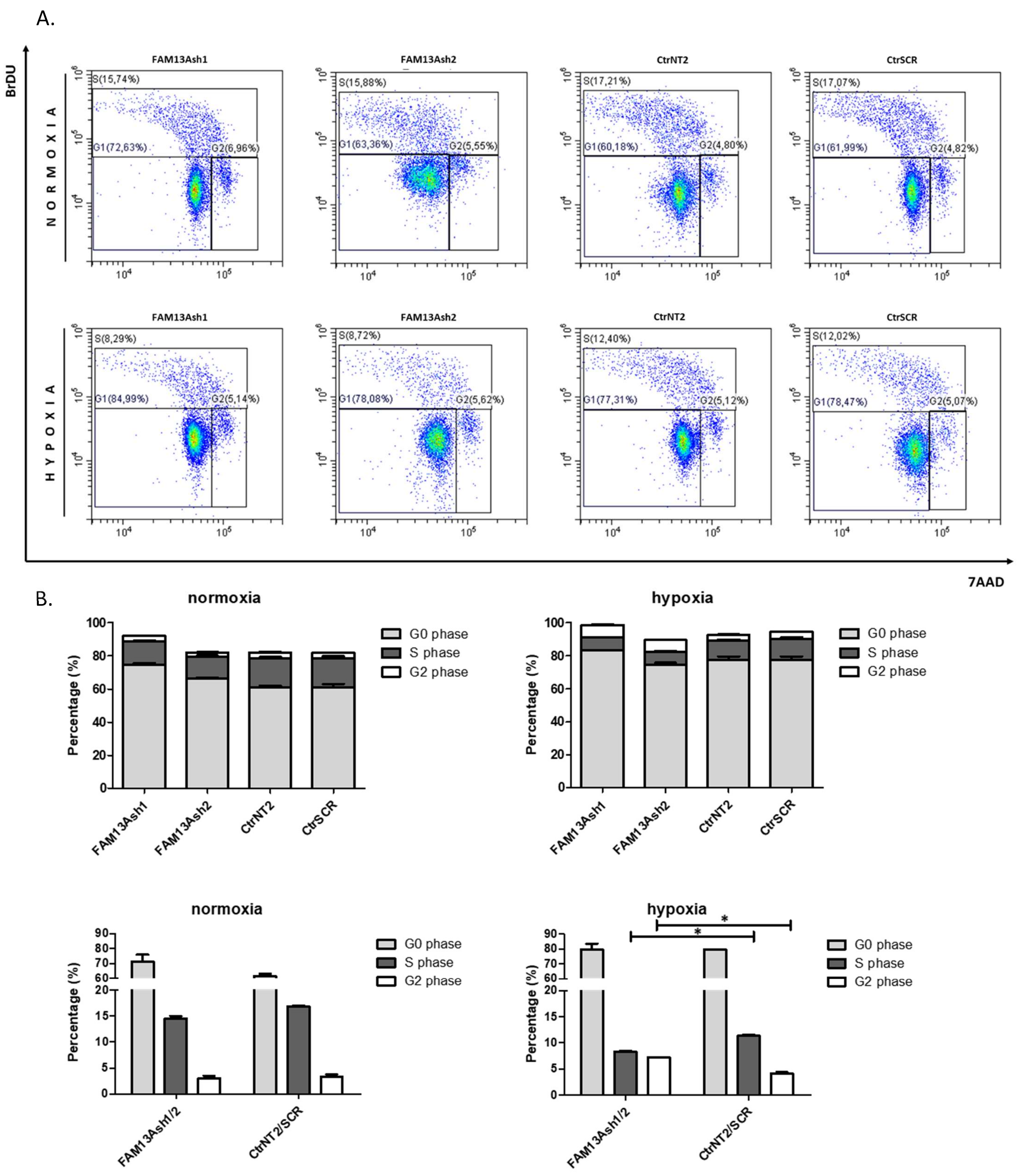

Figure S5. FAM13A depleted cells exhibit decreased proliferation. A. Representative flow cytometry plots of cell cycle analysis of A549 FAM13Ash1, FAM13Ash2 and controls (CtrNT2, CtrSCR) by BrdU and 7AAD staining**.** Scatter plots representing the percentages of the cells distribution (G1, G2, S stages). B. The bar chart represents the results of the cell cycle distribution. Unpaired Student's t-test indicates that percentages of cells in S and G2 phases are significantly different between FAM13Ash1/2 and both controls (CtrNT2/SCR) ($p < 0.05$; $p=0.0141$ and 0.0067, respctively). Bars represent mean ± SEM of 2 independent experiments.
